# The Diagnostic Value of Endobronchial Ultrasound-Guided Fine Needle Aspiration (EBUS-FNA) in Diagnosing FDG-PET-Avid Lymph Nodes in Extrapulmonary Malignancies

**DOI:** 10.7759/cureus.68269

**Published:** 2024-08-31

**Authors:** Mahreen Sana, Faheem Mahmood Butt, Adnan Amir

**Affiliations:** 1 Pulmonology, Shaukat Khanum Memorial Cancer Hospital and Research Centre, Lahore, PAK

**Keywords:** extra-pulmonary malignancies, mediastinal lymph nodes, avid lymph nodes, mediastinoscopy, hilar lymph nodes, pet-ct, ebus-fna

## Abstract

Background and objective

The accurate diagnosis of extrapulmonary malignancies with mediastinal lymphadenopathy is crucial for effective patient management. Endobronchial ultrasound-guided fine-needle aspiration (EBUS-FNA) has emerged as a valuable tool in assessing fluorodeoxyglucose (FDG)-positron emission tomography (PET)-avid lymph nodes (LNs). In this study, we aimed to evaluate the diagnostic value of EBUS-FNA in patients with mediastinal lymphadenopathy in extrapulmonary malignancies and compare its efficacy with PET-CT.

Methodology

This retrospective, cross-sectional study was conducted at Shaukat Khanum Memorial Cancer Hospital and Research Center, Lahore, from February 2018 to February 2023. It included patients with extrapulmonary malignancies with mediastinal lymphadenopathy displaying abnormal PET-CT uptake, with LN diameters ≥5 mm, excluding lung cancer cases. Data on demographics, malignancy type, LN involvement, PET-CT findings, and EBUS-FNA histopathology were collected. EBUS-FNA procedures involved a 22-gauge needle, and samples were analyzed cytologically and histologically. SPSS Statistics version 20 (IBM Corp., Armonk, NY) was used to perform the statistical analysis.

Results

The study analyzed a total of 216 patients. Males comprised 56.3% of the cohort, and females 43.7%. The most common malignancy was lymphoma (33.0%), followed by breast cancer (12.6%). EBUS-FNA exhibited a sensitivity of 90.9% compared to PET-CT's sensitivity of 72.7%. Lymph node morphology on EBUS showed low echogenicity and irregular borders in malignant cases. Subcarinal and right hilar were the most frequently sampled lymph nodes. The study found significant differences in lymph node characteristics between non-malignant and malignant groups, with EBUS-FNA effectively identifying malignancies.

Conclusions

EBUS-FNA demonstrates high sensitivity and diagnostic utility in identifying malignant lymph nodes in patients with extrapulmonary malignancies. Its effectiveness in detecting true positive cases highlights its importance as a complementary diagnostic tool to PET-CT in oncological diagnostics.

## Introduction

Cancer is a major health concern, accounting for almost one in six deaths (16.8%) and one in four deaths (22.8%) from noncommunicable diseases (NCDs) globally [1]. The National Lung Screening Trial (NLST) found that extrapulmonary malignancies accounted for 22.8% of deaths compared to 22.9% of deaths due to lung cancer, demonstrating that extrapulmonary malignancies are just as common and significant as lung cancers. These extrapulmonary neoplasms include thyroid, breast, kidney, liver, esophageal, pancreatic, and mediastinal tumors [2]. For both pulmonary and extrapulmonary malignancies, mediastinal staging is key for treatment planning, prognosis, and surgical eligibility. Conditions other than cancer, such as granulomatous diseases and pneumoconiosis, can cause mediastinal and hilar lymphadenopathy [3]. Determining a proper treatment plan requires an accurate diagnosis of mediastinal and hilar lymphadenopathy. 

Accurate nodal staging is crucial for effective cancer treatment and improved patient prognosis. Although lymph node (LN) excision with histological evaluation is a standard modality, it is intrusive and expensive [4,5]. Thoracic lesions have been sampled using various methods, including bronchial needle aspiration biopsy, percutaneous lung biopsy, mediastinoscopy, and thoracoscopy. While conventional transbronchial needle aspiration is safe, its viewing capabilities are limited. For peripheral thoracic lesions, percutaneous lung biopsy is suitable but not for mediastinal or hilar lesions. While mediastinoscopy and thoracoscopy are the gold standard for diagnosing mediastinal lesions, they require general anesthesia, cause significant trauma, are costly, and have higher postoperative complications [6-8]. Imaging techniques, including MRI, CT, positron emission tomography (PET), and lymphoscintigraphy, offer noninvasive evaluation of LNs throughout the body, potentially reducing the need for invasive procedures [9,10]. However, CT and PET-CT cannot provide a pathological diagnosis [6]. Advanced imaging techniques that assess biochemical and physiological activities at the cellular level are necessary for better nodal evaluation [11]. 

PET with 18F-FDG [2-(fluorine-18)fluoro-2-deoxy-d-glucose] improves tumor imaging and is mostly utilized for the staging of cancer, monitoring, and therapy response evaluation [12]. The combination of FDG-PET with CT enhances diagnostic accuracy by identifying FDG uptake and structural abnormalities [11]. Although FDG-PET is sensitive for detecting malignancy, FDG-avid lymph nodes are not definitively indicative of tumors and may occur in benign conditions, such as inflammation [13]. Increased avidity in PET-CT results often leads physicians to consider mediastinal lymph nodes as malignant. However false positive results are common with radiological examination [14,15]. Thus, while PET-CT helps narrow the differential diagnoses, it is insufficient to conclusively diagnose the cause of diffuse lymphadenopathy or investigate mediastinal lymph node metastasis [16].

A minimally invasive technique - the endobronchial ultrasound-guided needle aspiration (EBUS-FNA) - is employed for mediastinal lymph nodes, hilar lymph nodes, lung tumors in the area near the airway, and lung apex masses near the trachea [17]. It involves applying real-time ultrasound to identify critical areas and main blood vessels to keep away from and hence significantly improves the sampling accuracy and reduces the rate of bleeding. EBUS-FNA is an easy, minimally invasive, and relatively safe procedure [18,19]. Pulmonary, mediastinal masses, and undetermined mediastinal and hilar lymph node enlargement are diagnosed by the EBUS-FNA [20,21]. It can be applied in pulmonary and extrapulmonary tumors, staging and restaging carcinoma, and it provides sufficient tissue for molecular genotyping and immunohistochemistry, which is essential for determining treatment options including chemotherapy, targeted therapy, and re-biopsies once drug resistance develops [22,23]. 

The ability of EBUS-FNA to assess mediastinal or hilar metastasis from extrapulmonary malignancies has not been well established, despite the fact it has been used worldwide to diagnose lung cancer, sarcoidosis, tuberculosis, and lymphoma [14]. Mediastinoscopy is currently considered the gold standard for this purpose; however, it is invasive and has limitations in accessing certain lymph nodes. This technique is relatively new in Pakistan and is mainly offered in the diagnosis of lung cancers. Therefore, the purpose of this study was to examine the diagnostic value of EBUS-FNA in evaluating extrapulmonary cancers and to determine whether it can serve as a less invasive alternative to existing methods.

## Materials and methods

Study design and patients

This study was carried out at the Shaukat Khanum Memorial Cancer Hospital and Research Center in Lahore using a cross-sectional, retrospective design. Patients undergoing EBUS-FNA for mediastinal lymphadenopathy for extrapulmonary malignancies between February 2018 and February 2023 were included. The inclusion criteria were patients with extrapulmonary malignancies, mediastinal lymphadenopathy with aberrant PET-CT uptake, and lymph node diameters of at least 5 mm. Out of 826 patients who underwent EBUS-FNA, 216 fulfilled our inclusion criteria. Lung cancer patients were excluded from this study. Additionally, we excluded patients with incomplete data or insufficient information necessary for a comprehensive analysis.

Data collection

Data were recorded for parameters like patient demographics, the nature of the malignancy, the involvement of the mediastinal lymph node, the size and aberrant buildup of lymph nodes on PET-CT scans, and histopathological analysis of the EBUS-FNA samples.

Radiological evaluation

EBUS-FNA was suggested for lymph nodes with a transverse diameter of at least 5 mm that displayed abnormal uptake of FDG on PET-CT scan. A PET-CT scan was considered positive if it showed hypermetabolic activity consistent with malignancy. 

EBUS-FNA procedure

A pulmonologist used an EBUS-guided FNA EBUS (UC260FW) to execute all EBUS-FNA procedures. The procedure was conducted orally under local anesthesia with continuous monitoring of heart rate and oxygen saturation. A fine 22-gauge needle (model NA-201SX-4022; Olympus, Hamburg, Germany) was carefully used to penetrate each target nodal site at least twice, ensuring the retrieval of one or more samples of tissue core. EBUS-accessible lymph node stations were inspected before FNA. Cytology, cell blocks, and mycobacterial cultures were all performed using aspirated material. Additional aspiration was performed if necessary to obtain adequate tissue samples. Minor complications were recorded throughout the procedure. 

Pathological examination and final diagnosis

The slides were prepared by applying the aspirates to them and allowing them to air-dry. Subsequently, the aspirates were stained with hematoxylin and eosin (H&E) and fixed with 95% alcohol. The remaining aspirates were treated with a formalin-alcohol mixture for cell block preparation. The tissues were forwarded right away to the pathology division for histological and cytological analysis. The results of the EBUS-FNA procedure were categorized as malignant if the aspirated material contained cancer cells. 

Data analysis and statistical methods

SPSS Statistics version 20 (IBM Corp., Armonk, NY) was used to analyze the data, and descriptive statistics were computed. To maintain patient privacy, serial numbers were allocated to participants to ensure anonymity. According to generally recognized definitions, the diagnostic sensitivity, specificity, positive predictive value (PPV), negative predictive value (NPV), and overall accuracy of the PET-CT and EBUS-FNA scans were determined. Frequency distribution, chi-square tests, and t-tests were used to obtain statistical values.

## Results

Our analysis of 216 patients investigated for extrapulmonary cancers involved a broad overview of their features and results based on the diagnosis. This includes gender distribution, the type of malignancies, and the comparison of benign vs. malignant PET scan characteristics. The current study illustrates that EBUS-FNA of PET-avid lymph nodes provides diagnostic utility. This study highlights the important role of EBUS-FNA in the effective management and treatment of patients. 

Patient characteristics and clinical data

The distribution of gender and various types of malignancies among the 216 patients is shown in Table 1.

**Table 1:** Gender distribution and type of malignancies in the patients

Variable	Frequency	Percentage
Gender		
Male	121	56.3
Female	95	43.7
Type of malignancy		
Breast cancer	27	12.6
Cervix cancer	5	2.3
Hodgkin lymphoma	5	2.3
Leiomyosarcoma - left arm	1	0.5
Lymphoma	71	32.9
Nasopharyngeal cancer	6	2.8
Ovarian cancer	8	3.7
Adenocarcinoma - stomach	1	0.5
Adrenocortical tumor	1	0.5
Alveolus cancer	1	0.5
Anal canal cancer	1	0.5
Appendiceal carcinoma	1	0.5
Bladder cancer	5	2.3
Cholangicarcinoma	1	0.5
Colon cancer	6	2.8
Endometrioid carcinoma	2	0.9
Esophagus cancer	23	10.7
Gastric cancer	5	2.3
Gastroesophageal junction (GOJ) cancer	4	1.9
Melanoma	6	2.8
Myeloma	1	0.5
Pancreatic cancer	5	2.3
Penile cancer	1	0.5
Prostate cancer	4	1.9
Rectal cancer	6	2.8
Renal cell carcinoma	4	1.9
Right retroperitoneal cancer	1	0.5
Sarcoma	1	0.5
Skin cancer	2	0.9
Stomach cancer	4	1.9
Testicular cancer	2	0.9
Thyroid cancer	1	0.5
Tongue cancer	3	1.4
Total	216	100.0

Males comprised 56.3% (121 patients), while females accounted for 43.7% (95 patients). Lymphoma was the predominant malignancy: observed in 33.0% of the cases (71 patients). The incidence rate of breast cancer was 12.6% (n=27). Other significant malignancies included esophageal cancer (10.7%) and cancers of the nasopharynx, colon, rectum, and melanoma, each at 2.8%. 

Other malignancies, each constituting less than 1% of cases, included leiomyosarcoma of the left arm, adenocarcinoma of the stomach, adrenocortical tumor, alveolar cancer, anal canal cancer, appendiceal carcinoma, cholangiocarcinoma, myeloma, penile cancer, right retroperitoneal cancer, sarcoma, and thyroid cancer. These data underscore the diversity of malignancies diagnosed, with lymphoma and breast cancer being the most prevalent. 

Comparative analysis of PET scan characteristics in non-malignant vs. malignant patients

A total of 67 hilar and 148 mediastinal lymph nodes were sampled using EBUS-FNA following the PET scans. A comparative analysis between the non-malignant and malignant patient groups revealed significant differences in several characteristics, as shown in Table 2.

**Table 2:** Comparison of characteristics between non-malignant and malignant patients on PET scan PET-CT: positron emission tomography-computed tomography; SD: standard deviation; SUV: standardized uptake value

Characteristics	Non-malignant	Malignant	P-value
Gender			0.847
Female	26	74	
Male	31	84	
Age, years, mean ±SD	49 ±15	51 ±16	0.000
Size on PET-CT, mm, mean ±SD	16 ±12	17 ±10	0.000
SUV/abnormal uptake, mean ±SD	7.22 ±4.54	6.86 ±3.86	0.000

The gender distribution was similar between the groups, with non-malignant patients comprising 26 females and 31 males, and malignant patients comprising 84 males and 74 females (p=0.847). However, the age differed significantly between the groups: the mean age of non-malignant patients was 49 years (SD=15), while that of malignant patients was 51 years (SD=16) (p=0.000). The lymph node size on PET-CT also showed a significant difference, with non-malignant nodes averaging 16 cm (SD=12) and malignant nodes averaging 17 cm (SD=10) (p=0.000). Interestingly, standardized uptake value (SUV)/abnormal uptake was higher in non-malignant patients, with a mean of 7.22 (SD=4.54) compared to 6.86 (SD=3.86) in malignant patients (p=0.000). These results underscore the need for EBUS-FNA to overcome these diagnostic challenges and improve accuracy. 

Frequency of lymph nodes sampled via EBUS-FNA

The frequency of PET-avid lymph nodes sampled using EBUS-FNA after the PET scans is shown in Table 3.

**Table 3:** Frequency distribution of the lymph nodes sampled using EBUS-FNA EBUS-FNA: endobronchial ultrasound-guided fine-needle aspiration

Lymph node	Frequency
Subcarinal 7	78
Right hilar 10	40
Right lower paratracheal 4	35
Left hilar 10	27
Left lower paratracheal 4	10
Left upper paratracheal 2	8
Right upper paratracheal 2	12
Aortopulmonary 5	5

**Figure 1:**
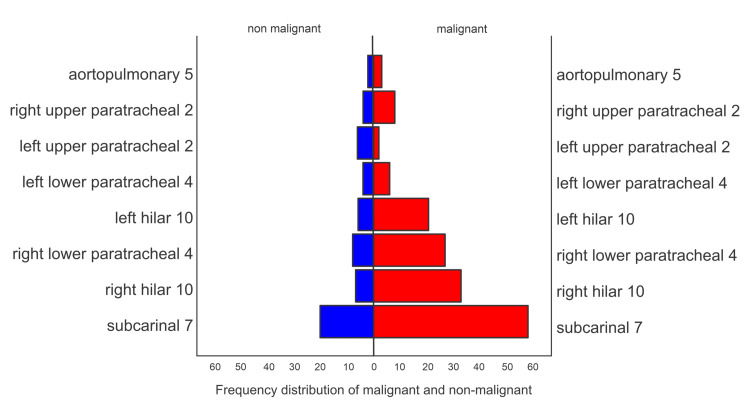
Frequency distribution of malignant and non-malignant lymph nodes

A total of 67 hilar and 148 mediastinal lymph nodes were identified. The subcarinal 7 node was most frequently sampled (78 instances), followed by the right hilar 10 (40 instances), right lower paratracheal 4 (35 instances), and left hilar 10 (27 instances). The other nodes sampled included left lower paratracheal 4 (10 instances), left upper paratracheal 2 (eight instances), right upper paratracheal 2 (12 instances), and aortopulmonary 5 (five instances). These findings emphasize the diagnostic priority of subcarinal, right hilar, and right lower paratracheal nodes, demonstrating the efficacy of EBUS-FNA in evaluating PET-avid lymph nodes for malignancy, as shown in Figure 1.

The final diagnosis of PET-avid lymph nodes with EBUS-FNA

The final diagnoses from the EBUS-FNA for PET-avid lymph nodes in extrapulmonary malignancies are presented in Table 4.

**Table 4:** Final diagnosis of extrapulmonary malignancies with EBUS-FNA EBUS-FNA: endobronchial ultrasound-guided fine-needle aspiration

Diagnosis	Frequency	Percentage
Lymphoma		
Classical Hodgkin lymphoma	2	0.9
Diffuse large B cell lymphoma	3	1.4
Hodgkin lymphoma	4	1.9
Hodgkin lymphoma, relapsed	5	2.3
Low-grade follicular lymphoma	1	0.5
Mantle cell lymphoma	1	0.5
Carcinoma		
Metastatic carcinoma	16	7.4
Metastatic adenocarcinoma	3	1.4
Metastatic breast carcinoma	5	2.3
Metastatic medullary carcinoma	1	0.5
Metastatic rectal carcinoma	1	0.5
Metastatic squamous cell carcinoma	2	0.9
Metastatic urothelial carcinoma	1	0.5
Reactive lymphadenopathy		
Reactive lymph node	93	43.1
Reactive lymph node with anthracosis	4	1.9
Reactive lymphoid hyperplasia	13	6.0
Inflammation		
Granulomatous inflammation	23	10.6
Necrotizing granulomatous inflammation	3	1.4
Non-necrotizing granulomatous inflammation	3	1.4
Others		
Anthracotic pigments	5	2.3
Atypical lymphoid infiltrate	5	2.3
Benign bronchial epithelial cells	4	1.9
Lymphoid cells, scattered histiocytes, alveolar macrophages	1	0.5
Necrotic debris	3	1.4
No malignant cells	4	1.9
Positive for malignant cells	1	0.5
Scanty tissue; mostly blood	1	0.5
Inadequate for cytological evaluation	7	3.2

Lymphomas included classical Hodgkin lymphoma (two cases, 0.9%), diffuse large B-cell lymphoma (DLBCL) (three cases, 1.4%), Hodgkin lymphoma (four cases, 1.9%), relapsed Hodgkin lymphoma (five cases, 2.3%), low-grade follicular lymphoma (one case, 0.5%), and mantle cell lymphoma (one case, 0.5%). Carcinomas included metastatic carcinoma (16 cases, 7.4%), adenocarcinoma (three cases, 1.4%), breast carcinoma (five cases, 2.3%), medullary carcinoma (one case, 0.5%), rectal carcinoma (one case, 0.5%), squamous cell carcinoma (two cases, 0.9%), and urothelial carcinoma (one case, 0.5%).

Apart from malignancies, reactive lymphadenopathy was the most common condition encountered (93 cases, 43.1%). Other diagnoses included reactive lymph nodes with anthracosis (four cases, 1.9%), reactive lymphoid hyperplasia (13 cases, 6.0%), granulomatous inflammation (23 cases, 10.6%), necrotizing granulomatous (three cases, 1.4%), non-necrotizing granulomatous (three cases, 1.4%), anthracotic pigments (five cases, 2.3%), atypical lymphoid infiltrate (five cases, 2.3%), benign bronchial epithelial cells (four cases, 1.9%), necrotic debris (three cases, 1.4%), and inadequate samples (seven cases, 3.2%). The data reveals that reactive lymphadenopathy was the most common diagnosis, followed by various forms of inflammation and metastatic carcinoma, underscoring the diverse range of diagnoses achieved through EBUS-FNA in the evaluation of extrapulmonary malignancies.

Characteristics of malignancies in PET scan and EBUS

**Figure 2:**
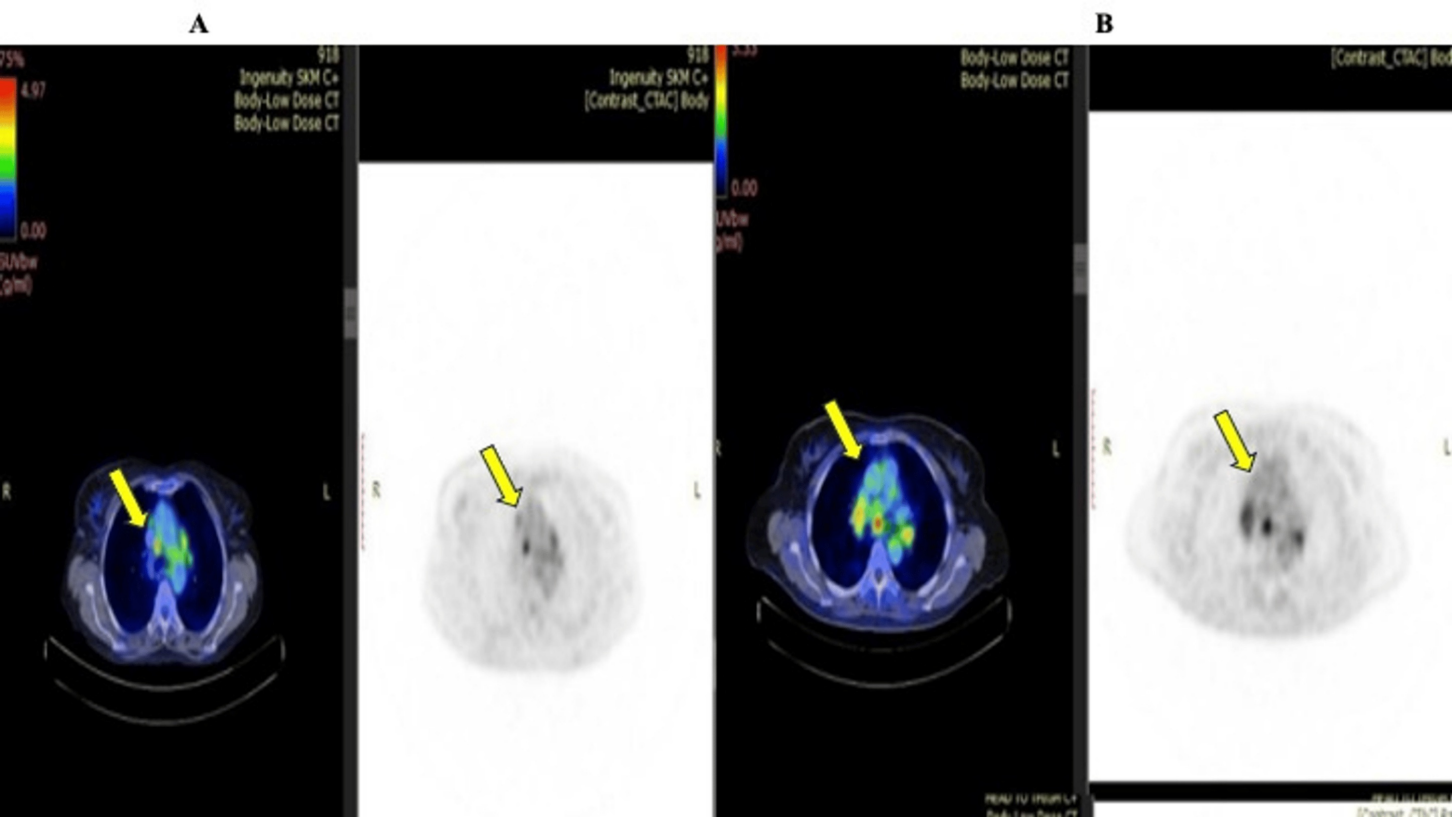
PET-CT findings in a patient with DLBCL and another with ovarian cancer A. PET-CT scan showing increased FDG uptake in the right lower paratracheal lymph node in a patient with DLBCL. B. PET-CT scan showing increased FDG uptake in multiple mediastinal and hilar lymph nodes in a patient with ovarian cancer DLBCL: diffuse large B-cell lymphoma; FDG: fluorodeoxyglucose; PET-CT: positron emission tomography-computed tomography

The PET scan revealed significant findings related to the involvement of lymph nodes in several patients. The increased FDG uptake at the right lower paratracheal lymph node of a DLBCL patient is shown in Figure 2A. This increased uptake suggests metabolic activity consistent with malignant infiltration. A patient with ovarian cancer demonstrated metastatic dissemination to several mediastinal and hilar lymph nodes by a wider range of nodal activity in these stations (Figure 2B). There was also increased FDG uptake in inflammation and non-malignant cases. This necessitated additional EBUS evaluation to confirm the malignant nature of PET-avid lymph nodes.

**Figure 3:**
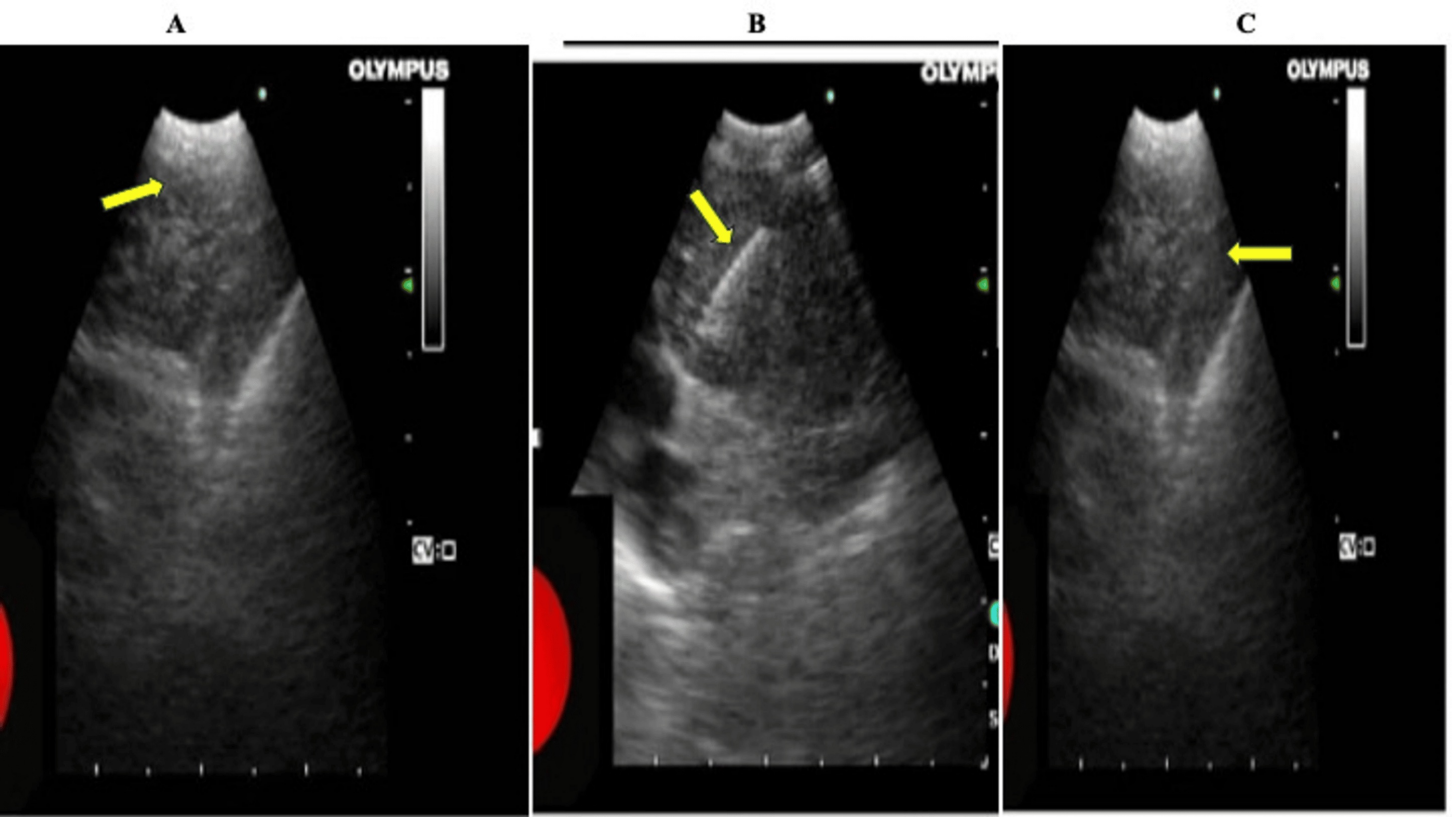
Representative endobronchial ultrasound images A. Endobronchial ultrasound image showing an enlarged subcarinal lymph node in a patient with renal cell carcinoma. B. Endobronchial ultrasound image depicting an FNA needle in a lymph node of a patient with Hodgkin lymphoma. C. Endobronchial ultrasound image displaying an enlarged subcarinal lymph node FNA: fine-needle aspiration

EBUS confirmed the final diagnosis of PET-avid lymph nodes in extrapulmonary malignancies and revealed the morphology of enlarged lymph nodes associated with different malignancies: high-grade renal cell carcinoma with low echogenicity, enlarged subcarinal lymph node, poorly margined and irregular in shape suggestive of potential malignancy (Figure 3A). In Hodgkin lymphoma, the lymph node displays malignant signs including round appearance and hypoechogenicity as shown in Figure 3B. Figure 3C shows a large subcarinal lymph node with hypoechoic features and sharp margins below the carina. The results suggest that EBUS is an effective tool for the identification and evaluation of lymph node involvement in different carcinomas.

## Discussion

This study evaluated gender distribution, types of malignancy, and diagnostic techniques to assess mediastinal and hilar lymphadenopathy in 216 patients. Our primary objective was to determine the diagnostic performance of EBUS-FNA in obtaining a pathological diagnosis from FDG-PET-avid lymph nodes in extrapulmonary malignant patients. Among various malignancies, lymphomas and breast cancers were the most common. PET scans showed significant differences between malignant and non-malignant groups, and EBUS-FNA demonstrated high diagnostic value in confirming these malignancies. This discussion will delve into the implications of these findings and emphasize the clinical utility of EBUS-FNA.

The sex distribution revealed a slightly higher prevalence of malignancies in males compared to females, aligning with epidemiological studies showing gender-related differences in cancer susceptibility and outcomes. The high prevalence of lymphoma and breast cancer highlights the need for targeted screening and preventive measures. Lymphomas, the most common malignancy in this group, represent a significant burden and require efficient diagnostic and therapeutic strategies. Our findings correlate with the reports of a well-recognized cancer database; GLOBOCAN, which provides statistical records and estimates of incidence and mortality from 185 countries for 36 types of cancers. It reports 79,990 new cases and 26,167 deaths from Hodgkin lymphoma and 509,590 new cases and 248,724 deaths from non-Hodgkin lymphoma (NHL) globally, with higher NHL rates in males (6.7 cases and 3.3 deaths per 100,000) compared to females (4.7 cases and 2.0 deaths per 100,000) [24]. The relatively high incidence of breast cancer aligns with global cancer statistics, underscoring the importance of raising awareness and early detection programs. The diversity of malignancies, including other cancers like leiomyosarcoma and cholangiocarcinoma, highlights the complexity of cancer diagnosis and the need for comprehensive diagnostic tools.

The significant differences in PET scans in terms of LN size and SUV or abnormal uptake between the non-malignant group and malignant group show that these parameters are very important when considering cancer diagnostics. The higher mean age in malignancy patients also indicates an increasing risk of malignancy with age. It has been suggested that the size of malignant LNs on PET-CT is an important indicator of tumor proliferation. Interestingly, the higher SUV/abnormal uptake in non-malignant cases highlights the potential of inflammatory conditions to mimic malignancy on PET scans. Studies have shown that elevated FDG uptake, seen in infections, inflammation, and cancer, complicates diagnosis, as FDG-PET-CT detects abnormalities but cannot distinguish between these conditions [25]. Concurrent CT imaging and comprehensive clinical information are crucial for accurate interpretation. This emphasizes the need for cautious interpretation of PET results and the complementary role of histopathological confirmation through procedures, such as EBUS-FNA.

The LN frequency distribution sampled via EBUS-FNA emphasizes the diagnostic importance of subcarinal, right hilar, and right lower paratracheal LNs. Frequent sampling of these nodes highlights their pivotal role in the diagnosis of suspected malignancies and other pathological conditions. As a minimally invasive procedure, EBUS-FNA offers real-time ultrasound guidance, enhancing sampling accuracy and safety, which is crucial for accurate diagnosis and staging of malignancies. The focus on these specific LNs reflects clinical practices that prioritize areas with a higher likelihood of pathological findings. This is consistent with a prior study that retrospectively reviewed 262 patients who had EBUS-TBNA from January 2016 to December 2023. The procedure was 96.9% diagnostic, indicating either mediastinal lymphadenopathy (83.2%) or a mass (16.8%). LN metastasis was most frequent in subcarinal and lower right paratracheal stations, with a rate of positivity of 54.6%. The complications were gentle and non-permanent. Literature has demonstrated that EBUS-TBNA is an accurate and minimally invasive diagnostic tool for the histologic diagnosis of lung cancer, mediastinal evaluation, and staging [26]. This suggests that EBUS-FNA is an effective modality that can safely sample crucial LNs, resulting in a higher yield for diagnostic and staging purposes of malignant diseases. 

In this study, the final diagnosis of PET-avid LNs in extrapulmonary malignant patients using EBUS-FNA revealed a predominance of reactive lymphadenopathy followed by several forms of inflammation and metastatic carcinoma. Due to the high incidence of reactive lymphadenopathy, most patients with benign conditions undergo EBUS-FNA, highlighting its role in the exclusion of malignancy. Additionally, granulomatous inflammation and other forms of inflammation demonstrate the multiple etiologies of lymphadenopathy. Finally, identifying metastatic carcinoma cases underlines EBUS-FNA’s role in the diagnosis and staging of metastatic disease. Another study analyzed 140 LNs from both EBUS-TBNA and PET scans of 79 patients and compared the methods and found that EBUS-TBNA significantly increased diagnostic accuracy and revealed more malignant LNs not noticed on PET. Among PET-positive LNs, 41.9% were malignant, and among PET-negative LNs, 74.1% showed reactive changes or granulomatous inflammation. Thus, EBUS-TBNA is vital in preventing false negatives of PET [27]. The diversity of diagnoses in this research shows that EBUS-FNA is instrumental in a comprehensive evaluation of lymphadenopathy in extrapulmonary malignancies.

The current study evaluated EBUS FNA for the pathological diagnosis of PET-avid lymph nodes in extrapulmonary malignancy and found a higher sensitivity of 90.9% for EBUS than that previously detected from PET (72.7%), showing superior efficacy in detecting true-positive cases. Results from a previous study, similar in concept to ours, suggest that the EBUS-TBNA, a minimally invasive method for investigation of mediastinal nodes, can downstage 40% of suspected stage III lung cancer cases disclosed by PET. Out of a total of 1176 samples from 486 patients examined at the Tampere University Hospital (2017-18), EBUS-TBNA had a diagnostic yield and accuracy rate of 89.5% and 77.9% respectively. The sensitivity for malignancy was 95.69% with a negative predictive value of 96.75%. Accordingly, EBUS is an extremely sensitive and successful method for detecting malignancies [28].

While our study provides a valuable contribution to the available data in our region, it has some limitations; these include its retrospective design, the fact that it was confined to a specified setting, and limited access to some lymph node stations. We recommend larger, prospective, and multicentric studies in the future to attain more comprehensive results and gain deeper insights.

## Conclusions

Our findings highlight the importance of EBUS-FNA as a precise and minimally invasive method for diagnosing FDG-PET-avid lymph nodes in patients with extrapulmonary malignancy. EBUS-FNA was highly sensitive in diagnosing malignant lymph nodes and distinguishing between nonmalignant conditions, which showed that it could be used as a diagnostic tool. These insights are critical for improving diagnostic accuracy, therapeutic decision-making, and patient outcomes in oncology. EBUS-FNA may obviate the need for more invasive surgical procedures, providing accurate diagnoses and supporting cost-effective patient care. To reach more comprehensive and insightful findings, future studies should adopt a prospective design and involve larger settings.
